# 1-Ethenyl-2-(methyl­sulfan­yl)-4,4-diphenyl-4,5-di­hydro-1*H*-imidazol-5-one (Thio­phenytoin analogue): synthesis, structure and Hirshfeld surface analysis

**DOI:** 10.1107/S2056989026002616

**Published:** 2026-03-17

**Authors:** Abderrazzak El Moutaouakil Ala Allah, Chiara Massera, Joel T. Mague, Abdulsalam Alsubari, Youssef Ramli

**Affiliations:** a Laboratory of Medicinal Chemistry Drug Sciences Research Center Faculty of Medicine and Pharmacy Mohammed V University in Rabat Morocco; b Dipartimento di Scienze Chimiche, della Vita e della Sostenibilità Ambientale, Università di Parma, Parco Area delle Scienze 17/A 43124 Parma, Italy; c Department of Chemistry Tulane University New Orleans LA 70118 USA; d Laboratory of Medicinal Chemistry, Faculty of Clinical Pharmacy, 21 September University, Yemen; Katholieke Universiteit Leuven, Belgium

**Keywords:** crystal structure, di­hydro­imidazolone, sulfan­yl, Hirshfeld surface

## Abstract

The title mol­ecule, C_18_H_16_N_2_OS, exhibits a slight ‘ruffling’ of the imidazolone ring, and the lone pair on the tricoordinate nitro­gen atom is involved in N→C π-bonding within the ring. Both the methyl group bonded to sulfur and the vinyl substituent lie very close to the plane of the five-membered ring. In the crystal, C—H⋯O hydrogen bonds form inversion dimers, which are linked into chains extending along the *b*-axis direction by C—H⋯π(ring) inter­actions. Hirshfeld surface analysis indicates that H⋯H inter­actions account for more than half of the inter­molecular contacts, with C—H⋯π(ring) inter­actions contributing a further quarter of the total.

## Abstract 2

The title compound C_18_H_16_N_2_OS has a slightly ruffled imidazolone ring with N–C π delocalization. In the crystal, C—H⋯O dimers link into *b*-axis chains *via* C—H⋯π contacts, with H⋯H inter­actions dominating the packing.

## 1. Chemical context

**Figure 1:**
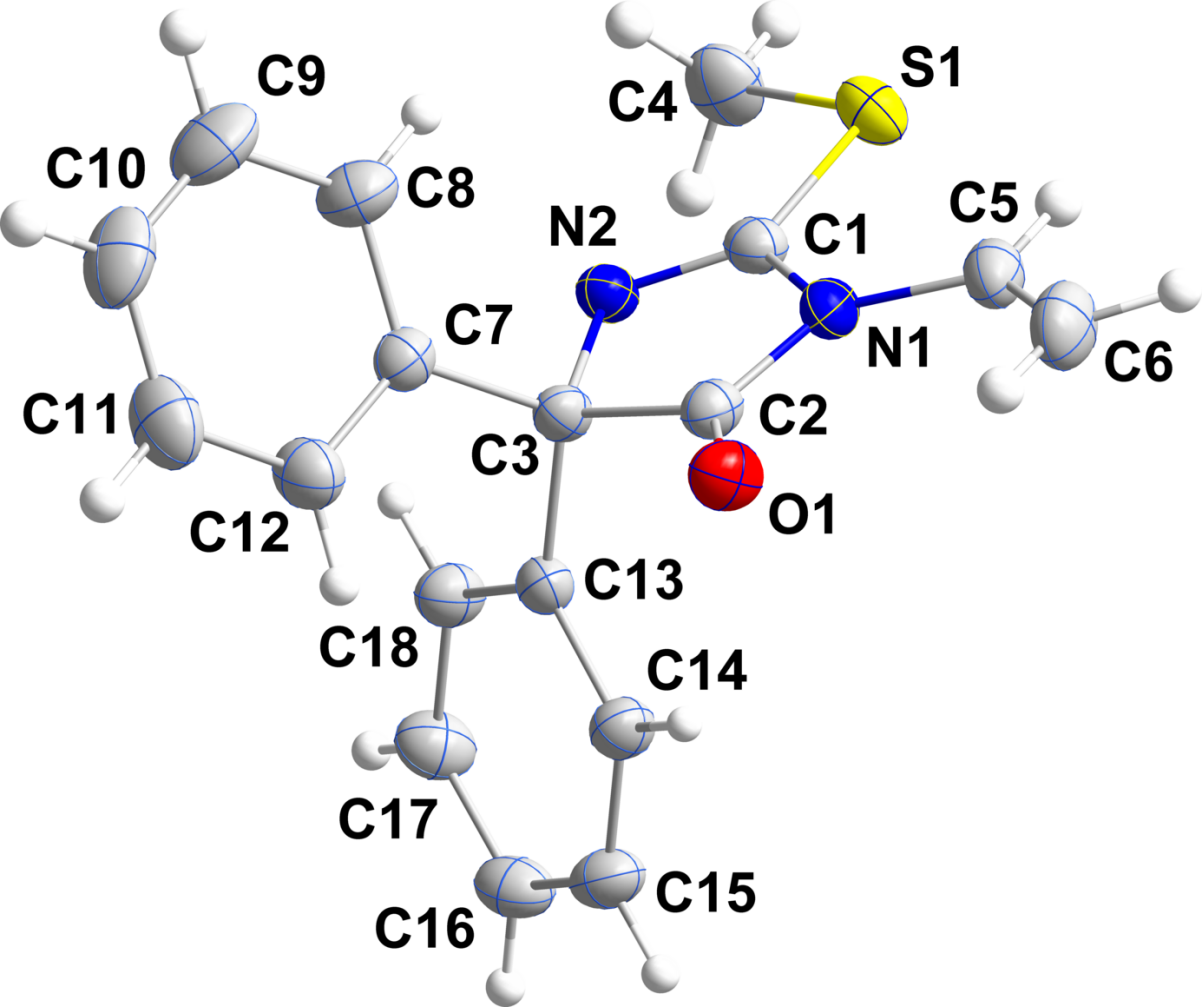
Perspective view of the title mol­ecule with labeling scheme and 30% probability ellipsoids.

Heterocycles incorporating both sulfur and nitro­gen atoms constitute a class of compounds of great inter­est in organic and medicinal chemistry, owing to the richness of their physicochemical properties and the diversity of their biological activities (El Moutaouakil Ala Allah *et al.*, 2024*a* ▸; Ettahiri *et al.*, 2024 ▸; Guerrab *et al.*, 2025 ▸). Among them, thio­hydantoin, a sulfur-containing heterocycle structurally related to hydantoin and characterized by the presence of a thioxo (C=S) group, has emerged as a privileged scaffold in medicinal chemistry (Gupta *et al.*, 2025 ▸). Its distinctive electronic and structural features enable a wide range of chemical modifications and promote strong inter­actions with various biological targets (El Moutaouakil Ala Allah *et al.*, 2024*b* ▸). Consequently, numerous thio­hydantoin derivatives have demonstrated significant pharmacological activities, including anti­diabetic (Ala Allah *et al.*, 2025*c* ▸), anti­cancer (Mezoughi *et al.*, 2021 ▸), and anti­microbial effects (El Moutaouakil Ala Allah *et al.*, 2024*c* ▸). In addition, some derivatives have shown promising performance as corrosion inhibitors (Ala Allah *et al.*, 2024 ▸; AlObaid *et al.*, 2024 ▸; El Moutaouakil Ala Allah *et al.*, 2024*d* ▸). In a continuation of our research on thio­hydantoin derivatives (Ramli *et al.*, 2017 ▸; Guerrab *et al.*, 2023*a*, ▸,*b* ▸, 2022 ▸; El Moutaouakil Ala Allah *et al.*, 2024*e* ▸), we report herein the synthesis of 2-(methyl­thio)-5,5-diphenyl-3-vinyl-3,5-di­hydro-4*H*-imidazol-4-one (Fig. 1 ▸) *via* an E2 elimination from 3-(2-bromo­eth­yl)-2-(methyl­thio)-5,5-diphenyl-3,5-di­hydro-4*H*-imidazol-4-one, a secondary halide, under conditions promoting unimolecular elimination in the presence of di­ethyl­amine [(Et)_2_NH] as the base and DMF as the solvent.
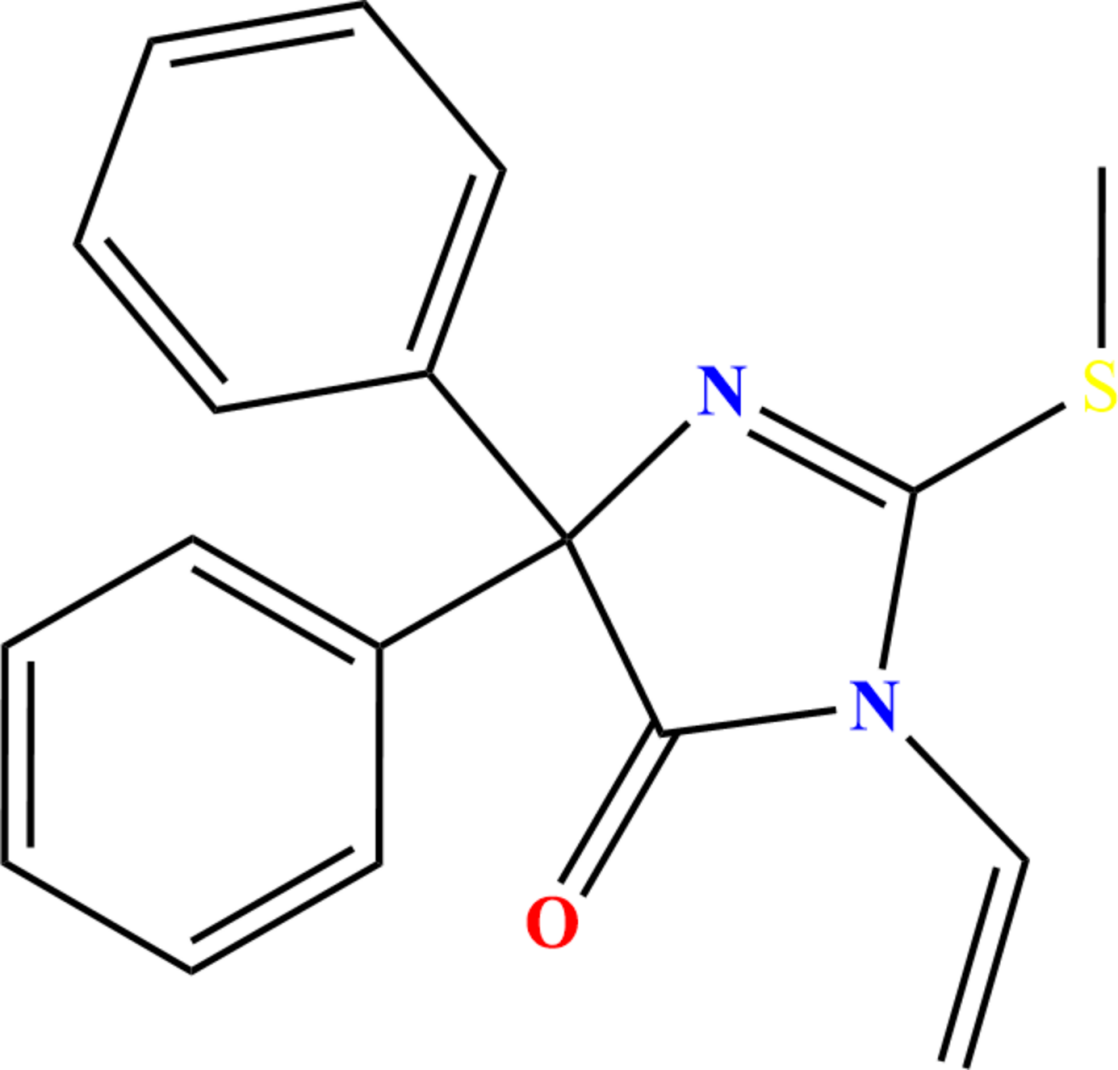


## 2. Structural commentary

The di­hydro­imidazolone ring is essentially planar, with a maximum deviation of 0.035 (1) Å from the mean plane (r.m.s. deviation of the fitted atoms = 0.001 Å). Atom C3 lies 0.035 (1) Å on one side of this plane, while C2 is displaced by 0.034 (1) Å on the opposite side. This slight out-of-plane displacement gives the ring a slight ‘ruffled’ conformation. The mean planes of the C7–C12 and C13–C18 rings are inclined to that of the di­hydro­imidazolone ring by 70.15 (9) and 66.01 (8)^o^, respectively. Atoms C4 and C6 both lie close to the plane of the di­hydro­imidazolone ring, as indicated by the C4—S1—C1—N2 and the C2—N1—C5—C6 torsion angles of −3.4 (2) and −1.8 (3)^o^, respectively. The sum of the bond angles about N1 is 360^o^ within experimental error, indicating that its lone pair is involved in N→C π-bonding. Although all the bonds to N1 are shorter than expected for formal single bonds, the N1—C2 distance of 1.387 (2) Å is the shortest, suggesting that the lone-pair inter­action is strongest in this bond.

## 3. Supra­molecular features

**Figure 2:**
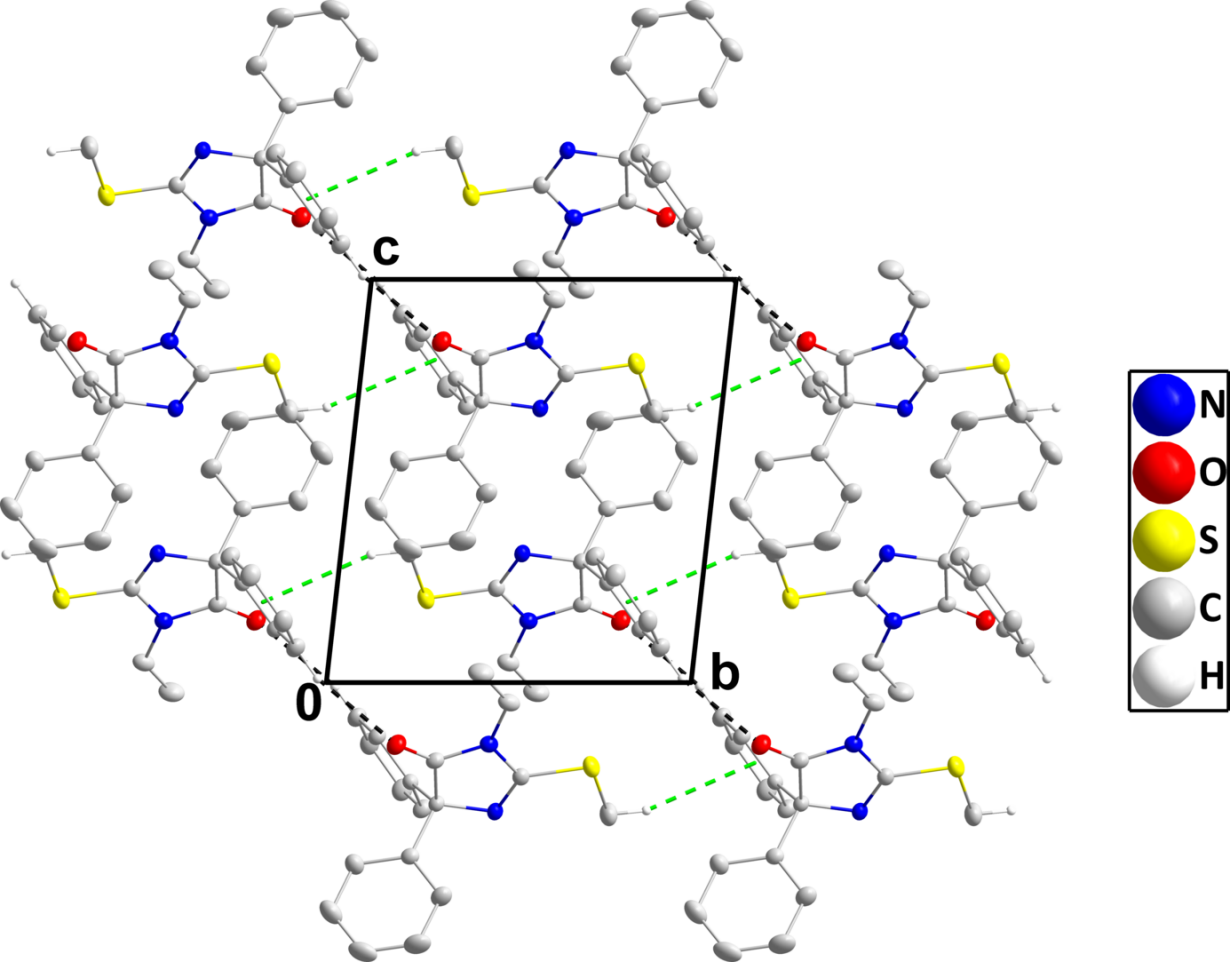
Packing viewed along the *a*-axis direction with C—H⋯O hydrogen bonds and C—H⋯π(ring) inter­actions depicted, respectively, by black and green dashed lines. Hydrogen atoms not involved in these inter­actions are omitted for clarity.

In the crystal, inversion dimers are generated by weak C15—H15⋯O1^ii^ hydrogen bonds and these are connected into chains extending along the *b*-axis direction by C4—H4*B*⋯C*g*3^i^ inter­actions (Table 1 ▸; C*g*3 is the centroid of the C13-C18 benzene ring). The chains pack with normal van der Waals contacts (Fig. 2 ▸).

## 4. Database survey

**Figure 3:**
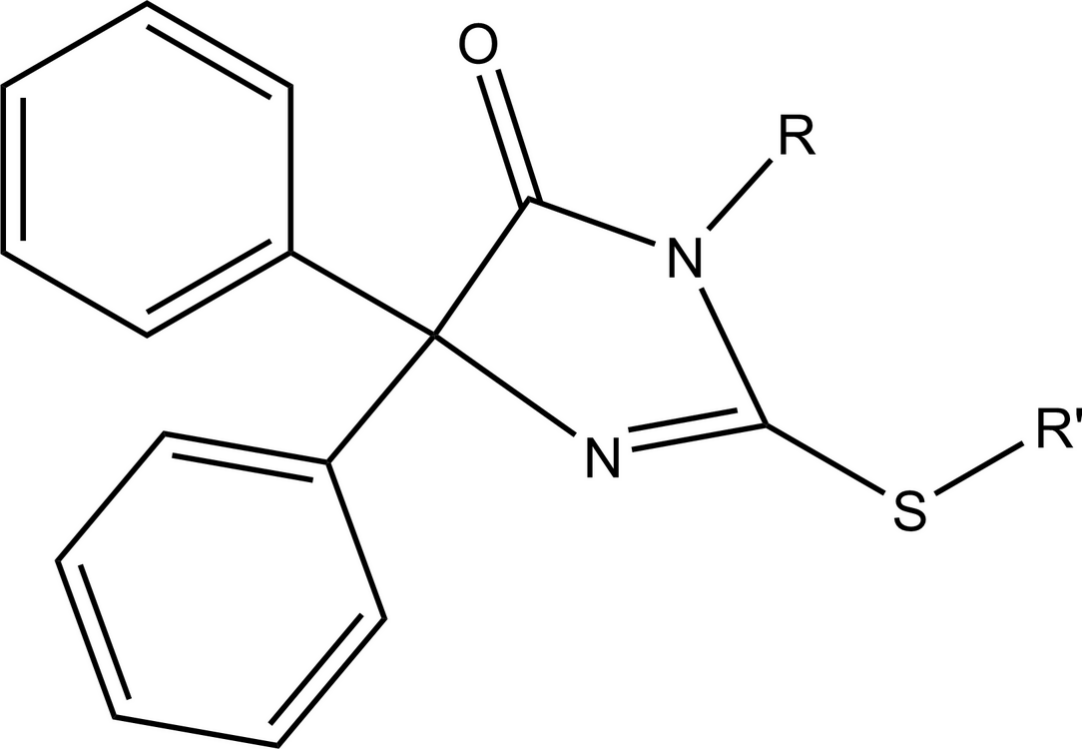
The search fragment used in the database survey.

A search of the Cambridge Structural Database (CSD, updated to January 2026; Groom *et al.*, 2016 ▸) with the search fragment shown in Fig. 3 ▸ (*R* = *R*′) gave nine hits, all of which are similar to the title compound. One group has *R* = *R*′ = Me (YEYYUA; El Moutaouakil Ala Allah *et al.*, 2023 ▸), Et (HOPQAI; El Moutaouakil Ala Allah *et al.*, 2024*a* ▸), *n*-Pr (RIJZIW; Akrad *et al.*, 2018 ▸) and benzyl (RAHGUF; Akrad *et al.*, 2017 ▸). In the second group, the nitro­gen and sulfur atoms are part of an exocyclic ring fused to the di­hydro­imidazolone ring, with *R*,*R*′ = –CH_2_CH_2_– (DIYRAE; Karolak-Wojciechowska *et al.*, 1985 ▸), –CH(CO_2_Et)CH_2_– (FURFED; Karolak-Wojciechowska & Kieć-Kononowicz, 1987 ▸), –(CH_2_)_3_– (IMTHZN; Kieć-Kononowicz *et al.*, 1981 ▸ and IMTHZN01; Guerrab *et al.*, 2019 ▸) and –(CH_2_)_2_O(CH_2_)_2_O(CH_2_)_2_O(CH_2_)_2_– (LIGWOR; Guerrab *et al.*, 2023*b* ▸). In all cases, the sum of the angles about the tri-coordinate nitro­gen atom in the five-membered ring is 360^o^ within experimental error, indicating participation of the nitro­gen lone pair N→C π-bonding. With the exception of DIYRAE, the N—C bond to the carbonyl carbon is the shortest of the three bonds involving this nitro­gen atom, as in the title compound; the corresponding distances range from 1.362 (2) Å in FURFED to 1.380 (2) Å in RAHGUF. In DIYRAE, the N—C bond to the carbonyl carbon is 1.383 (11) Å, whereas the other endocyclic N—C bond is 1.363 (11) Å. The reason for this variation is unclear; however, the associated standard uncertainties are relatively large, so the difference may not be significant. In all examples, the S—C bond corresponding to C4—S1 in the title mol­ecule lies close to the mean plane of the five-membered ring. The largest torsion angle (corresponding to the C4—S1—C1—N2 angle in the title mol­ecule) is C5—S1—C1—N1 in FURFED [−17.41 (15)^o^]. This is likely due to the constraints imposed by the ring attached to the di­hydro­imidazolone moiety. For *R* = Et, *n*-Pr and benzyl (Fig. 3 ▸), the carbon bonded to the nitro­gen atom lies in the plane of the di­hydro­imidazolone moiety, but the rest of the substituent is rotated well out of this plane. In the remaining derivatives, a similar twist is observed, but only to the extent allowed by the geometry of the pendant ring.

## 5. Hirshfeld surface analysis

**Figure 4:**
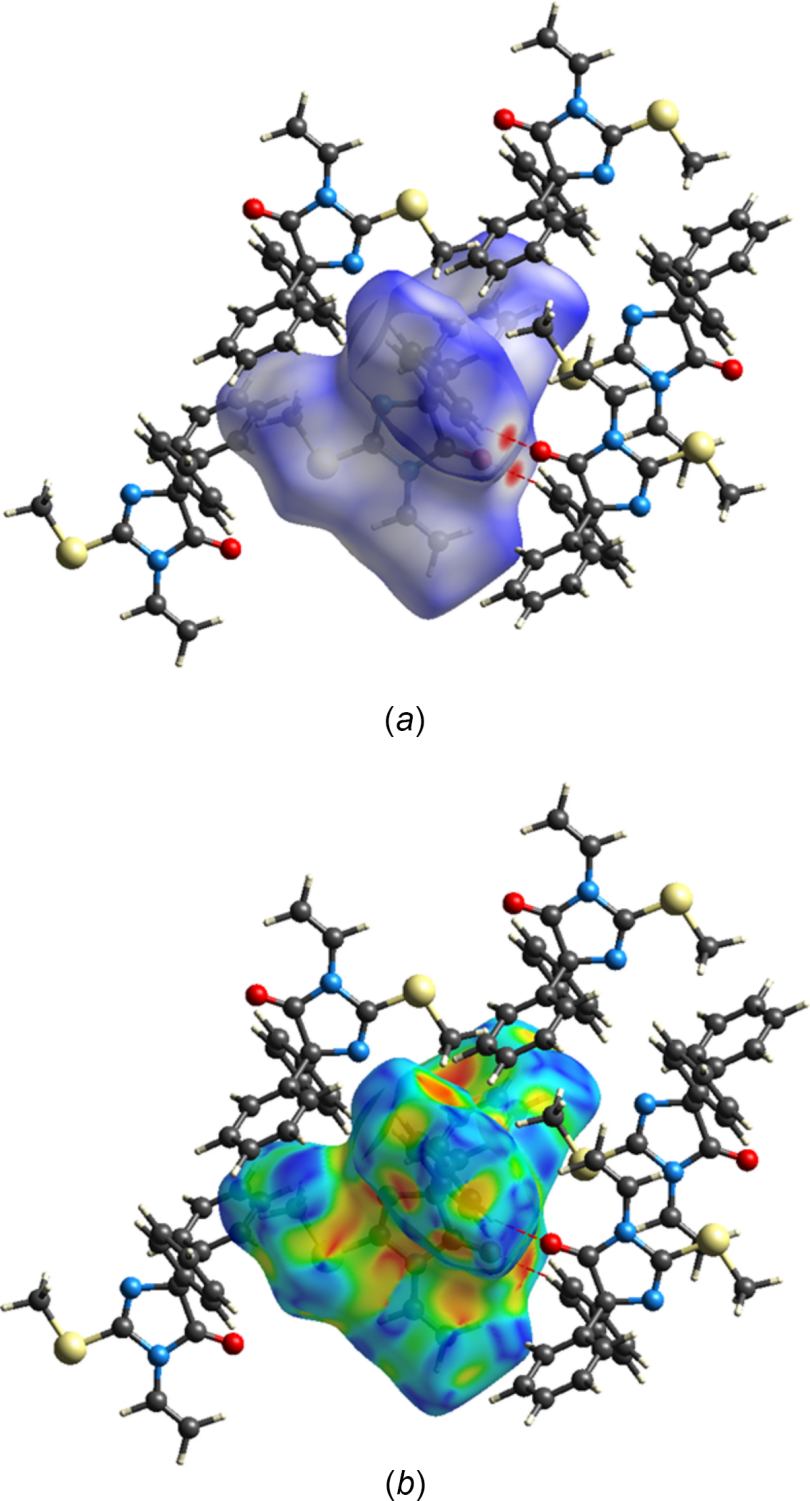
The Hirshfeld *d*_norm_ surface (*a*) and the Hirshfeld surface mapped over shape-index (*b*) showing several added neighboring mol­ecules.

**Figure 5:**
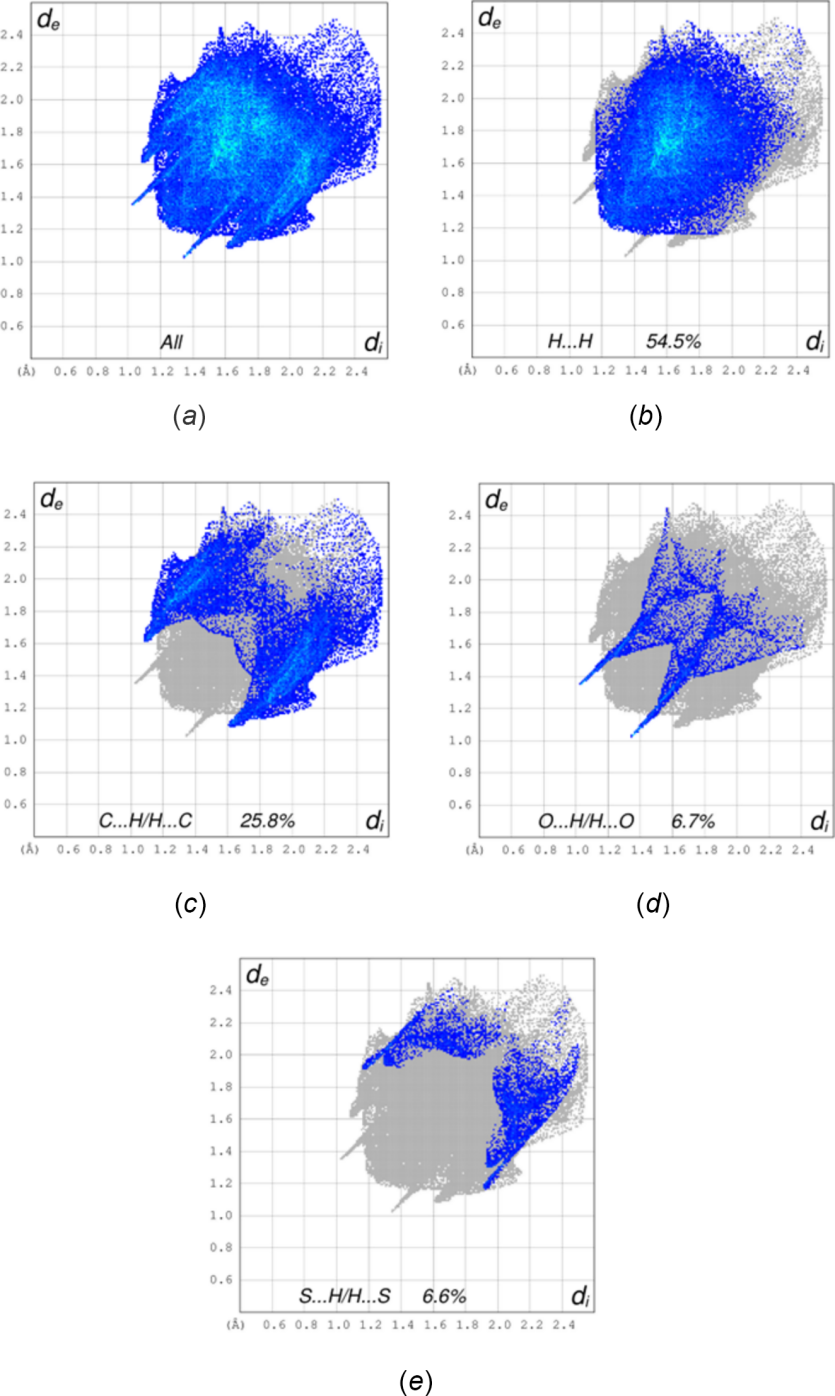
Two-dimensional fingerprint plots showing all inter­molecular contacts (*a*) and those limited to H⋯H contacts (*b*), C⋯H/H⋯C contacts (*c*), O⋯H/H⋯O contacts (*d*) and S⋯H/H⋯S contacts (*e*).

The Hirshfeld surface analysis of the inter­molecular inter­actions in the crystal of the title mol­ecule was performed with *CrystalExplorer* (Spackman *et al.*, 2021 ▸). Descriptions of the plots obtained and their inter­pretations have been previously published (Tan *et al.*, 2019 ▸). Fig. 4 ▸*a* shows the *d*_norm_ surface with several neighboring mol­ecules included. In the lower right of the figure, one of the inversion dimers formed by the C—H⋯O hydrogen bond listed in Table 1 ▸ (red dashed lines) is shown. In the upper right of the figure, the S—CH_3_ group forms a C—H⋯π(ring) inter­action with the phenyl group directly beneath it. Fig. 4 ▸*b* shows the Hirshfeld surface mapped over the shape-index function; the absence of orange triangle motifs indicates that significant π–π stacking inter­actions are not present. Fig. 5 ▸ presents the two-dimensional fingerprint plots for all inter­actions (*a*) and those limited to specific inter­action types. Fig. 5 ▸*b* shows that H⋯H inter­actions comprise 54.5% of the total, consistent with the mol­ecule’s periphery being dominated by hydrogen atoms. Next, contributing 25.8% of the total, are the C⋯H/H⋯C inter­actions (Fig. 5 ▸*c*). These appear as diffuse regions with a superimposed pair of blunt peaks. The peaks can be ascribed to the C—H⋯π(ring) inter­actions (Table 1 ▸), whereas the diffuse regions represent a range of van der Waals contacts. The C—H⋯O hydrogen bonds are reflected in the O⋯H/H⋯O inter­actions, which appear as a pair of relatively sharp peaks in Fig. 5 ▸*d* and comprise 6.7% of the total. Finally, the S⋯H/H⋯S inter­actions, which account for 6.6% of the total, appear in Fig. 5 ▸*e* as a pair of blunt peaks with a superimposed pair of sharper peaks. Although this might suggest C—H⋯S hydrogen bonding, the *d*_e_ + *d*_i_ distance of ≃ 3.1 Å indicates that it is simply a normal van der Waals contact.

## 6. Synthesis and crystallization

**Figure 6:**
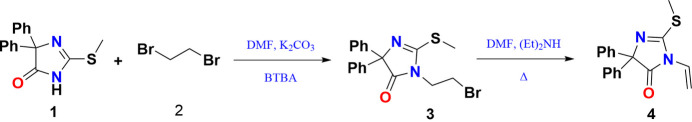
Synthesis of the title compound.

To a solution of 2-(methyl­thio)-5,5-diphenyl-3,5-di­hydro-4*H*-imidazol-4-one (**1**) (0.5 g, 1.2 mmol) in DMF (10 mL), half an equivalent of 1,2-di­bromo­ethane (**2**) (0.6 mmol) was added in the presence of K_2_CO_3_ (1.8 mmol) and a catalytic amount of benzyl­tri­butyl­ammonium bromide (BTBA, 10%). The reaction mixture was stirred at room temperature for 4h, as described in our previous work (El Moutaouakil Ala Allah *et al.*, 2023 ▸; El Moutaouakil Ala Allah *et al.*, 2025*b* ▸; El Moutaouakil Ala Allah *et al.*, 2024*c* ▸; El Moutaouakil Ala Allah *et al.*, 2025*a* ▸). The resulting compound (**3**) was subsequently refluxed in DMF in the presence of di­ethyl­amine, affording 2-(methyl­thio)-5,5-diphenyl-3-vinyl-3,5-di­hydro-4*H*-imidazol-4-one (**4**) *via* an elimination reaction (Fig. 6 ▸).

**2-(Methyl­thio)-5,5-diphenyl-3-vinyl-3,5-di­hydro-4*****H*****-imid­azol-4-one (4)** Yield = 68%; m.p. = 391–393K; Appearance: White powder; FT-IR (ATR, cm^−1^): 3062 (C—H Ar), 2985 (–CH_3_), 2852 (C—H Aliphatic), 1728 (C=O); ^1^H NMR (500 MHz, CDCl_3_): δ ppm 1.24 2.72 (*s*, 3H, S—CH_3_), 5.54 (*dd*, 2H, –CH_2_), 6.80 (*m*, 1H, CH), 7.20–7.30 (*m*, 10H, Ar H); ^13^C NMR (125 MHz, CDCl_3_); 14.18 (S—CH_3_), 76.20 (C—2Ph), 118.22 (–CH_2_), 130.14 (CH), 124.20, 126.23, 128.40, 140.62 (C—Ar), 162.57 (C=N), 178.12 (C=O).

## 7. Refinement details

Crystal data, data collection and structure refinement details are summarized in Table 2 ▸. The carbon-bound H atoms were placed in calculated positions and refined isotropically using the riding model, with C—H distances ranging from 0.93 to 0.99 Å and *U*_iso_(H) set to 1.2–1.5*U*_eq_(C).

## Supplementary Material

Crystal structure: contains datablock(s) I. DOI: 10.1107/S2056989026002616/vm2326sup1.cif

Structure factors: contains datablock(s) I. DOI: 10.1107/S2056989026002616/vm2326Isup2.hkl

Supporting information file. DOI: 10.1107/S2056989026002616/vm2326Isup3.cml

CCDC reference: 2536800

Additional supporting information:  crystallographic information; 3D view; checkCIF report

## Figures and Tables

**Table 1:** Hydrogen-bond geometry (Å, °) *Cg*3 is the centroid of the C13–C18 benzene ring.

*D*—H⋯*A*	*D*—H	H⋯*A*	*D*⋯*A*	*D*—H⋯*A*
C4—H4*B*⋯C*g*3^i^	0.96	2.90	3.815 (2)	160
C15—H15⋯O1^ii^	0.93	2.53	3.434 (2)	166

Symmetry codes: (i) 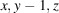; (ii) 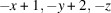.

**Table 2:** Experimental details

Crystal data	
Chemical formula	C_18_H_16_N_2_OS
*M*_r_	308.39
Crystal system, space group	Triclinic, *P* 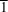
Temperature (K)	295
*a*, *b*, *c* (Å)	8.9481 (3), 9.0321 (2), 10.6253 (3)
α, β, γ (°)	81.333 (1), 69.520 (1), 82.227 (1)
*V* (Å^3^)	792.11 (4)
*Z*	2
Radiation type	Mo *K*α
μ (mm^−1^)	0.21
Crystal size (mm)	0.20 × 0.18 × 0.17

Data collection	
Diffractometer	Bruker D8 Venture PhotonIII
Absorption correction	Multi-scan (*SADABS*; Krause *et al.*, 2015 ▸)
*T*_min_, *T*_max_	0.723, 0.745
No. of measured, independent and observed [*I* > 2σ(*I*)] reflections	29667, 3209, 2950
*R*_int_	0.036
(sin θ/λ)_max_ (Å^−1^)	0.625

Refinement	
*R*[*F*^2^ > 2σ(*F*^2^)], *wR*(*F*^2^), *S*	0.041, 0.111, 1.04
No. of reflections	3209
No. of parameters	212
H-atom treatment	H atoms treated by a mixture of independent and constrained refinement
Δρ_max_, Δρ_min_ (e Å^−3^)	0.23, −0.41

Computer programs: *APEX5* (and *SAINT* (Bruker, 2016 ▸), *SHELXT2018/2* (Sheldrick, 2015*a* ▸), *SHELXL2019/2* (Sheldrick, 2015*b* ▸), *Mercury* (Macrae *et al.*, 2020 ▸), *WinGX* (Farrugia, 2012 ▸), *publCIF* (Westrip, 2010 ▸) and *enCIFer* (Allen *et al.*, 2004 ▸).

## supplementary crystallographic information

### 1-Ethenyl-2-(methylsulfanyl)-4,4-diphenyl-4,5-dihydro-1H-imidazol-5-one Crystal data

**Table:**

C_18_H_16_N_2_OS	*Z* = 2
*M**_r_* = 308.39	*F*(000) = 324
Triclinic, *P*1	*D*_x_ = 1.293 Mg m^−^^3^
*a* = 8.9481 (3) Å	Mo *K*α radiation, λ = 0.71073 Å
*b* = 9.0321 (2) Å	Cell parameters from 1561 reflections
*c* = 10.6253 (3) Å	θ = 2.1–26.0°
α = 81.333 (1)°	µ = 0.21 mm^−^^1^
β = 69.520 (1)°	*T* = 295 K
γ = 82.227 (1)°	Prismatic, colourless
*V* = 792.11 (4) Å^3^	0.20 × 0.18 × 0.17 mm

### 1-Ethenyl-2-(methylsulfanyl)-4,4-diphenyl-4,5-dihydro-1H-imidazol-5-one Data collection

**Table:**

Bruker D8 Venture PhotonIII diffractometer	3209 independent reflections
Radiation source: fine-focus sealed tube	2950 reflections with *I* > 2σ(*I*)
Graphite monochromator	*R*_int_ = 0.036
phi & ω scan	θ_max_ = 26.4°, θ_min_ = 2.1°
Absorption correction: multi-scan (SADABS; Krause *et al*., 2015)	*h* = −11→10
*T*_min_ = 0.723, *T*_max_ = 0.745	*k* = −11→10
29667 measured reflections	*l* = −13→13

### 1-Ethenyl-2-(methylsulfanyl)-4,4-diphenyl-4,5-dihydro-1H-imidazol-5-one Refinement

**Table:**

Refinement on *F*^2^	0 restraints
Least-squares matrix: full	Hydrogen site location: mixed
*R*[*F*^2^ > 2σ(*F*^2^)] = 0.041	H atoms treated by a mixture of independent and constrained refinement
*wR*(*F*^2^) = 0.111	*w* = 1/[σ^2^(*F*_o_^2^) + (0.055*P*)^2^ + 0.2157*P*] where *P* = (*F*_o_^2^ + 2*F*_c_^2^)/3
*S* = 1.04	(Δ/σ)_max_ < 0.001
3209 reflections	Δρ_max_ = 0.23 e Å^−^^3^
212 parameters	Δρ_min_ = −0.41 e Å^−^^3^

### 1-Ethenyl-2-(methylsulfanyl)-4,4-diphenyl-4,5-dihydro-1H-imidazol-5-one Special details

**Table:**

Geometry. All esds (except the esd in the dihedral angle between two l.s. planes) are estimated using the full covariance matrix. The cell esds are taken into account individually in the estimation of esds in distances, angles and torsion angles; correlations between esds in cell parameters are only used when they are defined by crystal symmetry. An approximate (isotropic) treatment of cell esds is used for estimating esds involving l.s. planes.

### 1-Ethenyl-2-(methylsulfanyl)-4,4-diphenyl-4,5-dihydro-1H-imidazol-5-one Fractional atomic coordinates and isotropic or equivalent isotropic displacement parameters (Å^2^)

**Table:**

	*x*	*y*	*z*	*U*_iso_*/*U*_eq_	
N1	0.30806 (14)	0.53530 (14)	0.15287 (12)	0.0456 (3)	
N2	0.41715 (14)	0.49764 (13)	0.32000 (12)	0.0437 (3)	
O1	0.21905 (14)	0.78849 (13)	0.15425 (12)	0.0576 (3)	
S1	0.42204 (5)	0.24750 (4)	0.20897 (5)	0.05768 (16)	
C1	0.38291 (16)	0.43747 (16)	0.23382 (14)	0.0438 (3)	
C2	0.28861 (16)	0.67747 (16)	0.19379 (14)	0.0441 (3)	
C3	0.37180 (16)	0.66082 (15)	0.30036 (13)	0.0403 (3)	
C4	0.5259 (3)	0.1856 (2)	0.3269 (2)	0.0708 (5)	
H4A	0.611516	0.247781	0.309316	0.106*	
H4B	0.569044	0.083047	0.317336	0.106*	
H4C	0.452827	0.192825	0.417262	0.106*	
C5	0.2620 (2)	0.4928 (2)	0.04959 (16)	0.0565 (4)	
C6	0.1893 (3)	0.5785 (3)	−0.0238 (2)	0.0745 (5)	
C7	0.25522 (16)	0.71697 (16)	0.43112 (14)	0.0437 (3)	
C8	0.1613 (2)	0.6187 (2)	0.53057 (18)	0.0634 (4)	
H8	0.169625	0.517770	0.518152	0.076*	
C9	0.0550 (2)	0.6700 (3)	0.6487 (2)	0.0846 (6)	
H9	−0.007131	0.602963	0.715446	0.101*	
C10	0.0403 (2)	0.8174 (3)	0.6681 (2)	0.0848 (7)	
H10	−0.031074	0.850703	0.748042	0.102*	
C11	0.1310 (2)	0.9168 (3)	0.5695 (2)	0.0788 (6)	
H11	0.120184	1.017900	0.582140	0.095*	
C12	0.2388 (2)	0.8667 (2)	0.45097 (19)	0.0614 (4)	
H12	0.300294	0.934462	0.384488	0.074*	
C13	0.52751 (16)	0.73904 (15)	0.24654 (13)	0.0401 (3)	
C14	0.56481 (18)	0.84435 (17)	0.13351 (15)	0.0482 (3)	
H14	0.491099	0.874571	0.088924	0.058*	
C15	0.7120 (2)	0.90521 (19)	0.08628 (17)	0.0578 (4)	
H15	0.736437	0.975100	0.009807	0.069*	
C16	0.82111 (19)	0.86263 (19)	0.15194 (19)	0.0597 (4)	
H16	0.919585	0.902990	0.120003	0.072*	
C17	0.7836 (2)	0.7593 (2)	0.26591 (19)	0.0612 (4)	
H17	0.856806	0.730985	0.311200	0.073*	
C18	0.63817 (19)	0.69766 (18)	0.31316 (16)	0.0525 (4)	
H18	0.614243	0.628148	0.389912	0.063*	
H6A	0.169 (3)	0.530 (3)	−0.092 (2)	0.090 (7)*	
H6B	0.151 (3)	0.684 (3)	−0.007 (3)	0.103 (8)*	
H5	0.293 (3)	0.386 (3)	0.034 (3)	0.103 (8)*	

### 1-Ethenyl-2-(methylsulfanyl)-4,4-diphenyl-4,5-dihydro-1H-imidazol-5-one Atomic displacement parameters (Å^2^)

**Table:**

	*U*^11^	*U*^22^	*U*^33^	*U*^12^	*U*^13^	*U*^23^
N1	0.0450 (6)	0.0517 (7)	0.0435 (6)	−0.0105 (5)	−0.0168 (5)	−0.0055 (5)
N2	0.0481 (6)	0.0381 (6)	0.0467 (6)	−0.0065 (5)	−0.0182 (5)	−0.0020 (5)
O1	0.0607 (7)	0.0557 (6)	0.0628 (7)	−0.0048 (5)	−0.0336 (5)	0.0049 (5)
S1	0.0605 (3)	0.0467 (2)	0.0703 (3)	−0.00652 (17)	−0.0231 (2)	−0.01581 (18)
C1	0.0400 (7)	0.0441 (7)	0.0459 (7)	−0.0090 (5)	−0.0112 (6)	−0.0040 (6)
C2	0.0416 (7)	0.0508 (8)	0.0410 (7)	−0.0099 (6)	−0.0157 (6)	0.0015 (6)
C3	0.0446 (7)	0.0379 (6)	0.0406 (7)	−0.0057 (5)	−0.0181 (6)	0.0001 (5)
C4	0.0841 (13)	0.0495 (9)	0.0846 (13)	0.0050 (9)	−0.0378 (11)	−0.0119 (9)
C5	0.0525 (9)	0.0746 (11)	0.0476 (8)	−0.0166 (8)	−0.0166 (7)	−0.0137 (7)
C6	0.0723 (12)	0.1042 (17)	0.0591 (11)	−0.0116 (11)	−0.0334 (9)	−0.0147 (11)
C7	0.0401 (7)	0.0504 (8)	0.0422 (7)	−0.0038 (6)	−0.0170 (6)	−0.0022 (6)
C8	0.0548 (9)	0.0658 (10)	0.0592 (10)	−0.0092 (8)	−0.0089 (8)	0.0032 (8)
C9	0.0618 (11)	0.1100 (18)	0.0605 (11)	−0.0099 (11)	0.0017 (9)	0.0029 (11)
C10	0.0556 (11)	0.130 (2)	0.0620 (11)	0.0081 (12)	−0.0082 (9)	−0.0347 (12)
C11	0.0651 (11)	0.0857 (14)	0.0867 (14)	0.0062 (10)	−0.0188 (10)	−0.0414 (12)
C12	0.0581 (9)	0.0581 (9)	0.0646 (10)	−0.0058 (7)	−0.0128 (8)	−0.0154 (8)
C13	0.0427 (7)	0.0388 (6)	0.0394 (6)	−0.0043 (5)	−0.0138 (5)	−0.0060 (5)
C14	0.0512 (8)	0.0468 (8)	0.0465 (7)	−0.0069 (6)	−0.0178 (6)	0.0004 (6)
C15	0.0584 (9)	0.0523 (9)	0.0549 (9)	−0.0137 (7)	−0.0101 (7)	0.0039 (7)
C16	0.0462 (8)	0.0554 (9)	0.0734 (11)	−0.0132 (7)	−0.0123 (8)	−0.0062 (8)
C17	0.0512 (9)	0.0627 (10)	0.0768 (11)	−0.0098 (7)	−0.0312 (8)	−0.0013 (8)
C18	0.0529 (8)	0.0545 (8)	0.0538 (8)	−0.0114 (7)	−0.0247 (7)	0.0047 (7)

### 1-Ethenyl-2-(methylsulfanyl)-4,4-diphenyl-4,5-dihydro-1H-imidazol-5-one Geometric parameters (Å, º)

**Table:**

N1—C2	1.3868 (19)	C8—C9	1.384 (3)
N1—C1	1.4082 (19)	C8—H8	0.9300
N1—C5	1.4170 (19)	C9—C10	1.360 (3)
N2—C1	1.2724 (18)	C9—H9	0.9300
N2—C3	1.4782 (17)	C10—C11	1.371 (4)
O1—C2	1.2068 (18)	C10—H10	0.9300
S1—C1	1.7452 (14)	C11—C12	1.387 (3)
S1—C4	1.792 (2)	C11—H11	0.9300
C2—C3	1.5386 (18)	C12—H12	0.9300
C3—C7	1.5267 (19)	C13—C14	1.385 (2)
C3—C13	1.5344 (18)	C13—C18	1.390 (2)
C4—H4A	0.9600	C14—C15	1.392 (2)
C4—H4B	0.9600	C14—H14	0.9300
C4—H4C	0.9600	C15—C16	1.372 (2)
C5—C6	1.291 (3)	C15—H15	0.9300
C5—H5	0.99 (3)	C16—C17	1.382 (3)
C6—H6A	0.98 (2)	C16—H16	0.9300
C6—H6B	0.99 (3)	C17—C18	1.382 (2)
C7—C12	1.381 (2)	C17—H17	0.9300
C7—C8	1.381 (2)	C18—H18	0.9300
			
C2—N1—C1	107.34 (11)	C7—C8—H8	119.9
C2—N1—C5	127.43 (14)	C9—C8—H8	119.9
C1—N1—C5	125.23 (14)	C10—C9—C8	120.7 (2)
C1—N2—C3	107.02 (11)	C10—C9—H9	119.6
C1—S1—C4	98.78 (8)	C8—C9—H9	119.6
N2—C1—N1	115.82 (13)	C9—C10—C11	119.80 (18)
N2—C1—S1	125.42 (11)	C9—C10—H10	120.1
N1—C1—S1	118.76 (11)	C11—C10—H10	120.1
O1—C2—N1	126.56 (13)	C10—C11—C12	120.1 (2)
O1—C2—C3	128.33 (14)	C10—C11—H11	120.0
N1—C2—C3	105.10 (11)	C12—C11—H11	120.0
N2—C3—C7	111.22 (11)	C7—C12—C11	120.39 (18)
N2—C3—C13	107.03 (11)	C7—C12—H12	119.8
C7—C3—C13	112.69 (11)	C11—C12—H12	119.8
N2—C3—C2	104.32 (11)	C14—C13—C18	118.84 (13)
C7—C3—C2	109.81 (11)	C14—C13—C3	123.49 (12)
C13—C3—C2	111.43 (11)	C18—C13—C3	117.64 (12)
S1—C4—H4A	109.5	C13—C14—C15	120.39 (14)
S1—C4—H4B	109.5	C13—C14—H14	119.8
H4A—C4—H4B	109.5	C15—C14—H14	119.8
S1—C4—H4C	109.5	C16—C15—C14	120.35 (15)
H4A—C4—H4C	109.5	C16—C15—H15	119.8
H4B—C4—H4C	109.5	C14—C15—H15	119.8
C6—C5—N1	126.82 (19)	C15—C16—C17	119.49 (15)
C6—C5—H5	119.8 (15)	C15—C16—H16	120.3
N1—C5—H5	113.4 (15)	C17—C16—H16	120.3
C5—C6—H6A	115.8 (14)	C18—C17—C16	120.60 (15)
C5—C6—H6B	121.4 (15)	C18—C17—H17	119.7
H6A—C6—H6B	123 (2)	C16—C17—H17	119.7
C12—C7—C8	118.82 (15)	C17—C18—C13	120.31 (14)
C12—C7—C3	120.90 (13)	C17—C18—H18	119.8
C8—C7—C3	120.26 (14)	C13—C18—H18	119.8
C7—C8—C9	120.17 (19)		
			
C3—N2—C1—N1	−3.01 (16)	N2—C3—C7—C8	−23.74 (18)
C3—N2—C1—S1	176.66 (10)	C13—C3—C7—C8	−143.93 (14)
C2—N1—C1—N2	−1.21 (16)	C2—C3—C7—C8	91.21 (16)
C5—N1—C1—N2	178.98 (13)	C12—C7—C8—C9	−1.1 (3)
C2—N1—C1—S1	179.09 (9)	C3—C7—C8—C9	−179.58 (16)
C5—N1—C1—S1	−0.71 (19)	C7—C8—C9—C10	0.5 (3)
C4—S1—C1—N2	−3.39 (15)	C8—C9—C10—C11	0.4 (3)
C4—S1—C1—N1	176.27 (12)	C9—C10—C11—C12	−0.8 (3)
C1—N1—C2—O1	−174.50 (14)	C8—C7—C12—C11	0.7 (3)
C5—N1—C2—O1	5.3 (2)	C3—C7—C12—C11	179.19 (16)
C1—N1—C2—C3	4.63 (14)	C10—C11—C12—C7	0.2 (3)
C5—N1—C2—C3	−175.57 (13)	N2—C3—C13—C14	130.00 (14)
C1—N2—C3—C7	123.87 (12)	C7—C3—C13—C14	−107.43 (15)
C1—N2—C3—C13	−112.64 (12)	C2—C3—C13—C14	16.55 (18)
C1—N2—C3—C2	5.56 (14)	N2—C3—C13—C18	−47.95 (16)
O1—C2—C3—N2	172.92 (14)	C7—C3—C13—C18	74.61 (16)
N1—C2—C3—N2	−6.19 (13)	C2—C3—C13—C18	−161.41 (13)
O1—C2—C3—C7	53.65 (19)	C18—C13—C14—C15	1.2 (2)
N1—C2—C3—C7	−125.46 (12)	C3—C13—C14—C15	−176.70 (14)
O1—C2—C3—C13	−71.94 (18)	C13—C14—C15—C16	−0.6 (2)
N1—C2—C3—C13	108.96 (12)	C14—C15—C16—C17	−0.3 (3)
C2—N1—C5—C6	−1.8 (3)	C15—C16—C17—C18	0.7 (3)
C1—N1—C5—C6	177.93 (17)	C16—C17—C18—C13	−0.1 (3)
N2—C3—C7—C12	157.80 (14)	C14—C13—C18—C17	−0.9 (2)
C13—C3—C7—C12	37.61 (18)	C3—C13—C18—C17	177.16 (15)
C2—C3—C7—C12	−87.25 (16)		

### 1-Ethenyl-2-(methylsulfanyl)-4,4-diphenyl-4,5-dihydro-1H-imidazol-5-one Hydrogen-bond geometry (Å, º)

*Cg*3 is the centroid of the C13–C18 benzene ring.

**Table:**

*D*—H···*A*	*D*—H	H···*A*	*D*···*A*	*D*—H···*A*
C4—H4*B*···C*g*3^i^	0.96	2.90	3.815 (2)	160
C15—H15···O1^ii^	0.93	2.53	3.434 (2)	166

Symmetry codes: (i) *x*, *y*−1, *z*; (ii) −*x*+1, −*y*+2, −*z*.
